# Venous thromboembolism in patients hospitalized for knee joint replacement surgery

**DOI:** 10.1038/s41598-020-79490-w

**Published:** 2020-12-31

**Authors:** Karsten Keller, Lukas Hobohm, Stefano Barco, Irene Schmidtmann, Thomas Münzel, Martin Engelhardt, Lukas Eckhard, Stavros V. Konstantinides, Philipp Drees

**Affiliations:** 1 Department of Cardiology, Cardiology I, University Medical Center Mainz (Johannes Gutenberg-University Mainz), Langenbeckstr. 1, 55131 Mainz, Germany; 2 Center for Thrombosis and Hemostasis (CTH), University Medical Center Mainz (Johannes Gutenberg-University Mainz), Mainz, Germany; 3 Clinic for Angiology, University Hospital Zurich, Zurich, Switzerland; 4 Institute of Medical Biostatistics, Epidemiology and Informatics (IMBEI), University Medical Center Mainz (Johannes Gutenberg-University Mainz), Mainz, Germany; 5 German Center for Cardiovascular Research (DZHK), Partner Site Rhine Main, Germany; 6 Department for Orthopaedics, Trauma Surgery and Hand Surgery, Klinikum Osnabrück, Osnabrück, Germany; 7 Institute for Applied Training Science, Leipzig, Germany; 8 Department of Orthopaedics and Traumatology, University Medical Center Mainz (Johannes Gutenberg-University Mainz), Mainz, Germany; 9 Department of Cardiology, Democritus University of Thrace, Alexandroupolis, Greece

**Keywords:** Epidemiology, Outcomes research, Cardiology

## Abstract

Patients undergoing knee joint replacement (KJR) are at high risk of postoperative venous thromboembolism (VTE), but data on the time trends of VTE rate in this population are sparse. In this analysis of the German nationwide inpatient sample, we included all hospitalizations for elective primary KJR in Germany 2005–2016. Overall, 1,804,496 hospitalized patients with elective primary KJR (65.1% women, 70.0 years [IQR 63.0–76.0]) were included in the analysis. During hospitalization, VTE was documented in 23,297 (1.3%) patients. Total numbers of primary KJR increased from 129,832 in 2005 to 167,881 in 2016 (β-(slope)-estimate 1,978 [95% CI 1,951 to 2,004], P < 0.001). In-hospital VTE decreased from 2,429 (1.9% of all hospitalizations for KJR) to 1,548 (0.9%) cases (β-estimate − 0.77 [95% CI − 0.81 to − 0.72], P < 0.001), and in-hospital death rate from 0.14% (184 deaths) to 0.09% (146 deaths) (β-estimate − 0.44 deaths per year [95% CI − 0.59 to − 0.30], P < 0.001). Infections during hospitalization were associated with a higher VTE risk. VTE events were independently associated with in-hospital death (OR 20.86 [95% CI 18.78–23.15], P < 0.001). Annual number of KJR performed in Germany increased by almost 30% between 2005 and 2016. In parallel, in-hospital VTE rates decreased from 1.9 to 0.9%. Perioperative infections were associated with higher risk for VTE. Patients who developed VTE had a 21-fold increased risk of in-hospital death.

## Introduction

Knee joint replacement (KJR) surgery is one of the most common orthopedic procedures currently performed in the ageing populations of Western countries^1^. In the year 2010, the number of KJR procedures performed in the United States increased to more than 700,000 per year^2^. It has been foreseen that this number will further grow to approximately 3.5 million KJR procedures by the year 2030^2^. Although this operation is one of the most successful and life-changing interventions available today^1^ and can substantially improve mobility and quality of life, these patients are characterized by high risk for perioperative venous thromboembolism (VTE), presenting as deep vein thrombosis and/or pulmonary embolism (PE), even in the era of established pharmacological thromboprophylaxis^1–6^. The release of thromboplastins from the dissected soft tissue and reamed bone, as well as venous stasis both initiated during surgery and the postoperative immobility, provoke a high rate of VTE events^1^.

Without thromboprophylaxis, the rate of proximal deep vein thrombosis after KJR surgery was reported to be between 5 and 22%, that of PE of 1.5–10%^2,6,7^. This risk can be reduced substantially by the use of pharmacological thromboprophylaxis and with a three-month cumulative incidence of symptomatic VTE falling to 2.3%^2,8^.

With increasing numbers of KJR procedures in western countries^2,6,9–11^, the number of VTE events is expected to rise^2,6^. Actual data about temporal trends regarding total numbers of performed KJR and particularly the impact of VTE on in-hospital mortality of KJR patients is sparse. This knowledge for optimizing secondary prevention strategies in this patient population. Thus, the aims of our study were to investigate, (a) temporal trends in the total numbers of KJR performed in Germany between 2005 and 2016, including trends in the patients’ age as well as comorbidities; (b) the total burden and temporal trends of VTE complications following KJR; (c) the impact of VTE on mortality rates in KJR patients; and (d) predictors of VTE during hospitalization.

## Results

A total of 1,804,496 hospitalizations were recorded for elective (primary) KJR in Germany from 2005 to 2016 and were included in the present analysis (see Fig. S1 in the supplementary material for study flow chart). Among these, the majority of patients hospitalized (65.1%) were women, and 53.4% were 70 years or older (median 70.0 years [IQR 63.0–76.0]). The estimated average crude annual incidence of elective primary KJR was 221.0 KJR per 100,000 population-years during this time period.

### Temporal trends of primary surgical knee joint replacements, patients’ baseline characteristics and in-hospital events

**Figure 1:**
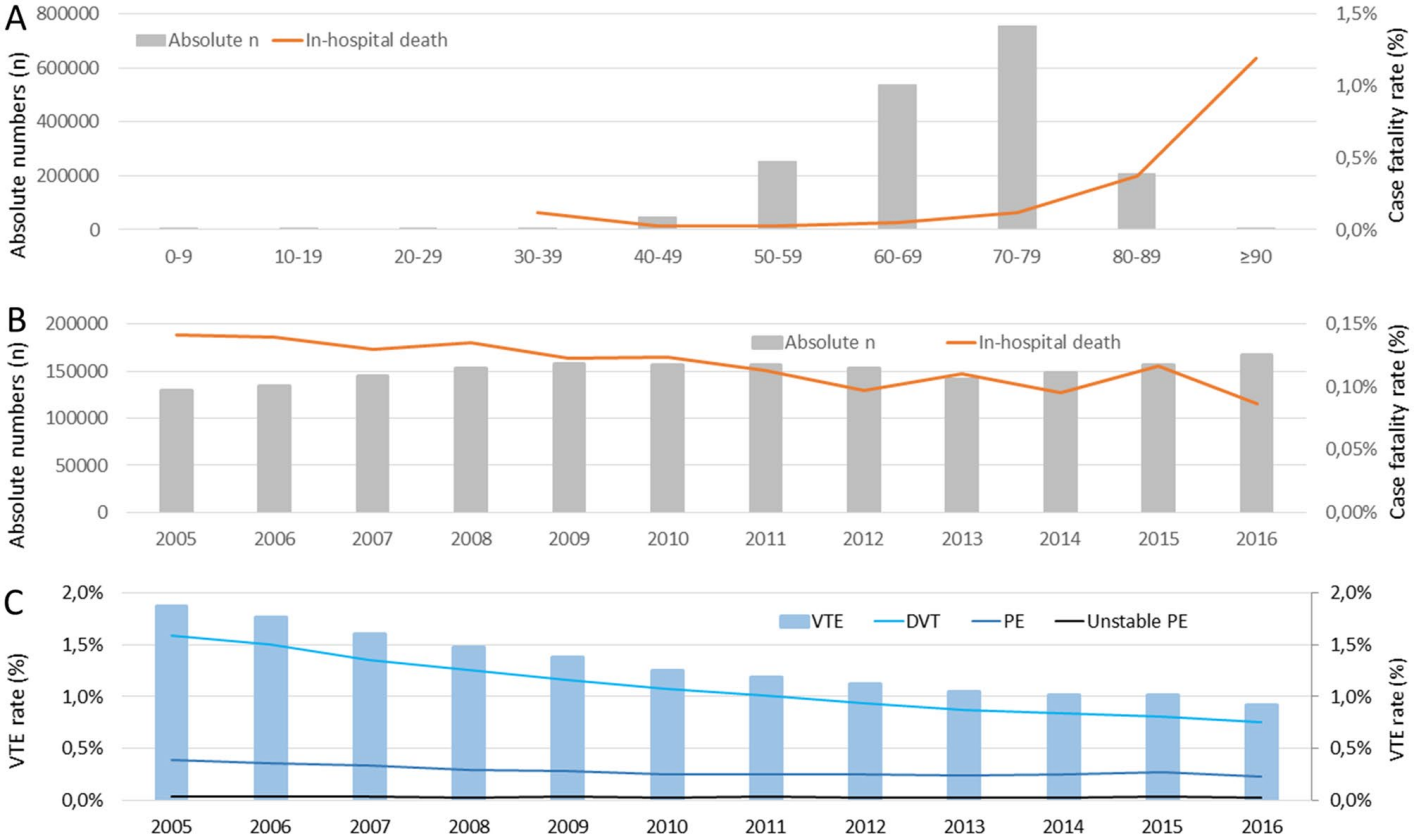
Time trends in absolute number of hospitalizations, in-hospital mortality and VTE rate in patients undergoing elective primary KJR in Germany from 2005 to 2016. (**A**) Total numbers of patients undergoing elective primary KJR (grey bars) and in-hospital death rate (orange line), stratified per decade of age, over the entire period 2005–2016 (cumulative data). (**B**) Annual total numbers of patients undergoing elective primary KJR (grey bars) and corresponding annual in-hospital death rates (orange line) in Germany in the period 2005–2016. (**C**) Percentage of patients undergoing elective primary HJR who developed in-hospital VTE (light blue bars); the panel further shows percentage of deep vein thrombosis and thrombophlebitis (DVT, light blue line), pulmonary embolism (PE, admiral blue line), and high-risk PE with hemodynamic instability (dark blue line).

The total number of KJR performed as well as in-hospital death rates increased substantially with age (Fig. 1A). While men were more frequently represented than women in the first three decades of life, the proportion of women was higher among patients aged 30 years and older (Fig. S2C in the supplementary material).

**Figure 2:**
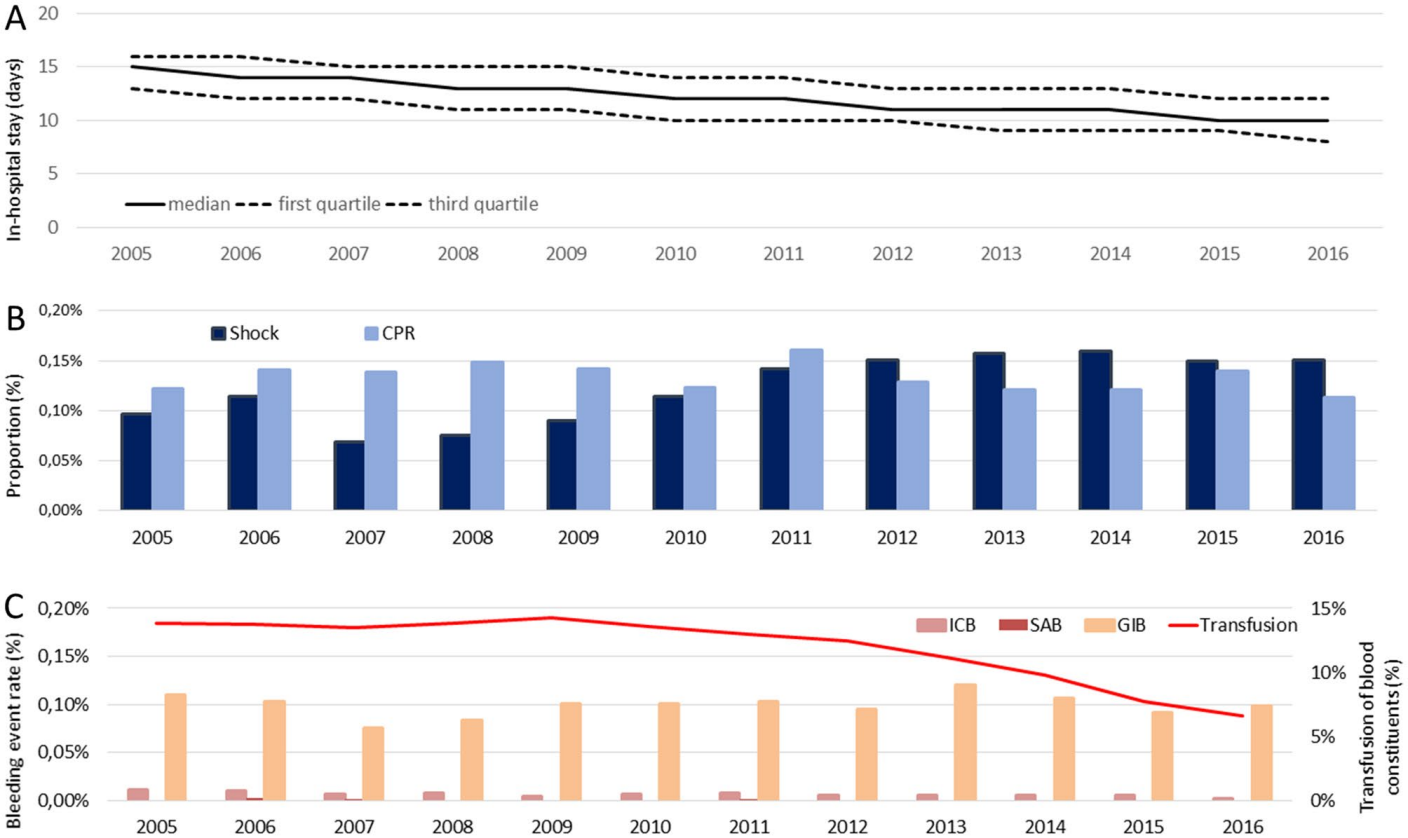
Time trends in length of hospitalization and rates of serious complications in patients undergoing elective primary KJR in Germany from 2005 to 2016. (**A**) Median duration of hospitalization (solid black line) with 25% and 75% IQR (dashed black lines). (**B**) Percentage of patients developing shock (dark blue bars) and undergoing cardio-pulmonary resuscitation (CPR, light blue bars) out of all patients undergoing elective KJR. (**C**) Percentage of patients with bleeding events such as intracerebral bleeding (ICB, light red bars), subarachnoid bleeding (SAB, dark red bars) and gastrointestinal bleeding (GIB, orange bars) as well as necessitation for transfusions of blood constituents (red line) out of all patients undergoing elective KJR.

The total number of patients who underwent KJR in Germany increased from 129,832 in the year 2005 to 167,881 in 2016 (β-(slope)-estimate 1,978 [95% CI 1,951 to 2,004] increase per year, P < 0.001). A total of 2,112 patients undergoing elective primary KJR died during the in-hospital stay for an overall perioperative death rate of 0.1%. The crude annual in-hospital death rate decreased from 0.14% (184 deaths) to 0.09% (146 deaths) of KJR-related hospitalizations (β-estimate − 0.4 deaths per year [95% CI − 0.6 to − 0.3], P < 0.001) over the time period studied (Fig. 1B, Table S2 in the supplementary material). The median duration of hospitalization for KJR decreased from 15 (13–16) days in 2005 to 10 (8–12) in 2016 (β-estimate − 0.12 [95% CI − 0.12 to − 0.12], P < 0.001) (Fig. 2A) and was primarily depending on patients’ age (Fig. S3 in the supplementary material).

The proportion of patients aged 70 years or older among those who underwent KJR, decreased from 54.0% in 2005 to 50.3% in 2016 (β-estimate − 0.14 [95% CI − 0.15 to − 0.13], P < 0.001). In parallel, the prevalence of some important comorbidities such as cancer and coronary artery disease decreased during the same timeframe (Fig. S2B + C, Fig. S4 and Table S1 in the supplementary material). Although females outweighed male patients with regard to KJR performed throughout the entire study period, the proportion of male patients increased slightly over time (Fig. S2A and Table S1 in the supplementary material). The rate of myocardial infarction, stroke, intracerebral bleeding complications as well as necessity of transfusions of blood constituents decreased (Fig. 2B + C, Fig. S5 and Table S2 in the supplementary material).

### Thromboembolic complications in patients undergoing elective knee joint replacement

A VTE event was recorded in 23,297 (1.3%) patients during hospitalization, presenting as deep venous thrombosis or thrombophlebitis (DVT) in 19,535 (83.9%) and of PE in 5,040 (21.6%) cases. Importantly, haemodynamic instability was recorded in 10.9% of the patients who suffered acute PE in-hospital. VTE events and consistently its subentities decreased over time. While 2,429 (1.9%) patients operated for KJR suffered VTE in hospital in the year 2005, only 1,548 (0.9%) VTE were recorded in 2016 (β-estimate − 0.77 [95% CI − 0.81 to − 0.72], P < 0.001). The rate of DVT (1.6% in 2005 to 0.8% in 2016; β-estimate − 0.82 [95% CI − 0.87 to − 0.77], P < 0.001) and PE (0.4% in 2005 to 0.2% in 2016; β-estimate − 0.52 [95% CI − 0.61 to − 0.42], P < 0.001) were reduced by almost 50% (Fig. 1 and Table S2 in the supplementary material). The number of VTE events increased with the patients’ age (β 0.14 [0.12 to 0.15], P < 0.001) (Fig. 1A).

Patients undergoing primary KJR who suffered VTE during hospitalization were more often women (72.5% vs. 65.0%, P < 0.001), older (72.0 [65.0–77.0] vs. 70.0 [63.0–76.0] year, P < 0.001), and more often obese (27.9% vs. 22.9%, P < 0.001) compared to those without VTE (Table 1). Typical risk factors for thrombosis such as cancer (1.4% vs. 0.8%, P < 0.001) and thrombophilia (whenever sought and detected; 0.3% vs. 0.1%, P < 0.001), were more prevalent in patients with VTE. Patients with VTE more frequently revealed cardiovascular, renal and respiratory comorbidities (Table 1). In-hospital infections such as pneumonia (2.8% vs. 0.3%, P < 0.001), urinary tract infections (5.2% vs. 2.8%, P < 0.001), and sepsis (0.26% vs. 0.04%, P < 0.001) all were more often recorded in association with VTE. Finally, myocardial infarction (0.6% vs. 0.2%, P < 0.001) and stroke (0.6% vs. 0.2%, P < 0.001) also were more frequently reported in patients with VTE (Table 1). The rate of infectious complications remained largely unchanged over time (Figures S5 as well as Table S2 in the supplementary material). At multivariable analysis, cancer, heart failure as well as infections such as sepsis, pneumonia, and urinary tract infection were independently associated with VTE events during the hospital stay (Table 2).

**Table 1:** Patient characteristics of 1,804,496 patients with elective KJR.

Parameters	Patients without VTE (n = 1,781,199; 98.7%)	Patients with VTE (n = 23,297; 1.3%)	P-value
Age	70.0 (63.0–76.0)	72.0 (65.0–77.0)	< 0.001
Age ≥ 70 years	950,241 (53.3%)	13,806 (59.3%)	< 0.001
Female gender *	1,156,887 (65.0%)	16,894 (72.5%)	< 0.001
In-hospital stay (days)	12 (10–14)	14 (12–18)	< 0.001
Obesity	407,073 (22.9%)	6,507 (27.9%)	< 0.001
**Comorbidities**			
Cancer	14,279 (0.8%)	336 (1.4%)	< 0.001
Coronary artery disease	162,093 (9.1%)	2,650 (11.4%)	< 0.001
Chronic heart failure	99,546 (5.6%)	2,340 (10.0%)	< 0.001
Peripheral artery disease	14,900 (0.8%)	283 (1.2%)	< 0.001
Atrial fibrillation/flutter	103,603 (5.8%)	1,954 (8.4%)	< 0.001
Chronic obstructive pulmonary disease	62,079 (3.5%)	1,122 (4.8%)	< 0.001
Essential arterial hypertension	1,058,340 (59.4%)	14,101 (60.5%)	0.001
Acute and chronic renal failure	99,552 (5.6%)	1,955 (8.4%)	< 0.001
Diabetes mellitus	305,510 (17.2%)	4,164 (17.9%)	0.004
Thrombophilia	1,810 (0.1%)	67 (0.3%)	< 0.001
**In-hospital conditions/outcomes**			
In-hospital death	1,590 (0.1%)	522 (2.2%)	< 0.001
Pneumonia	5,448 (0.3%)	649 (2.8%)	< 0.001
Sepsis	651 (0.04%)	60 (0.26%)	< 0.001
Urinary tract infection	50,497 (2.8%)	1,206 (5.2%)	< 0.001
Shock	2,008 (0.1%)	214 (0.9%)	< 0.001
Cardio-pulmonary resuscitation	1,915 (0.1%)	492 (2.1%)	< 0.001
Myocardial infarction	3,139 (0.2%)	147 (0.6%)	< 0.001
Stroke	2,752 (0.2%)	147 (0.6%)	< 0.001
Intracerebral bleeding	108 (0.01%)	8 (0.03%)	< 0.001
Gastro-intestinal bleeding	1,704 (0.1%)	77 (0.3%)	< 0.001
Transfusion of blood constituents	209,484 (11.8%)	4,849 (20.8%)	< 0.001

*Data available for 1,804,428 patients.

**Table 2:** Association of patients’ characteristics and in-hospital events with occurrence of VTE (univariate and multivariable logistic regression models).

	Univariate regression model		Multivariable regression model*	
	OR (95% CI)	P-value	OR (95% CI)	P-value
Age	1.02 (1.01–1.02)	< 0.001	1.01 (1.01–1.01)	< 0.001
Age ≥ 70 years	1.27 (1.24–1.31)	< 0.001	1.19 1.16–1.22)	< 0.001
Female gender *	1.42 (1.38–1.47)	< 0.001	1.40 (1.36–1.45)	< 0.001
Obesity	1.31 (1.27–1.35)	< 0.001	1.31 (1.27–1.35)	< 0.001
**Comorbidities**				
Cancer	1.81 (1.62–2.02)	< 0.001	1.82 (1.63–2.03)	< 0.001
Coronary artery disease	1.28 (1.23–1.34)	< 0.001	1.15 (1.11–1.20)	< 0.001
Chronic heart failure	1.89 (1.81–1.97)	< 0.001	1.53 (1.46–1.61)	< 0.001
Peripheral artery disease	1.46 (1.30–1.64)	< 0.001	1.29 (1.15–1.46)	< 0.001
Atrial fibrillation/flutter	1.48 (1.42–1.55)	< 0.001	1.26 (1.20–1.32)	< 0.001
Chronic obstructive pulmonary disease	1.40 (1.32–1.49)	< 0.001	1.24 (1.17–1.32)	< 0.001
Essential arterial hypertension	1.05 (1.02–1.08)	0.001	0.95 (0.93–0.98)	< 0.001
Acute and chronic renal failure	1.55 (1.48–1.62)	< 0.001	1.26 (1.20–1.33)	< 0.001
Diabetes mellitus	1.05 (1.02–1.09)	0.004	0.95 (0.92–0.99)	0.005
Thrombophilia	2.84 (2.22–3.62)	< 0.001	2.83 (2.22–3.62)	< 0.001
**In-hospital conditions**				
Pneumonia	9.34 (8.60–10.14)	< 0.001	7.21 (6.61–7.85)	< 0.001
Sepsis	7.06 (5.42–9.20)	< 0.001	5.16 (3.94–6.75)	< 0.001
Urinary tract infection	1.87 (1.77–1.98)	< 0.001	1.57 (1.48–1.66)	< 0.001
Myocardial infarction	3.60 (3.05–4.25)	< 0.001	2.61 (2.20–3.09)	< 0.001
Stroke	4.10 (3.47–4.85)	< 0.001	3.50 (2.96–4.14)	< 0.001

*Adjusted for age, sex, obesity, cancer, coronary artery disease, heart failure, atrial fibrillation/flutter, arterial hypertension, chronic obstructive pulmonary disease, acute and chronic renal failure, and diabetes mellitus.

**Figure 3:**
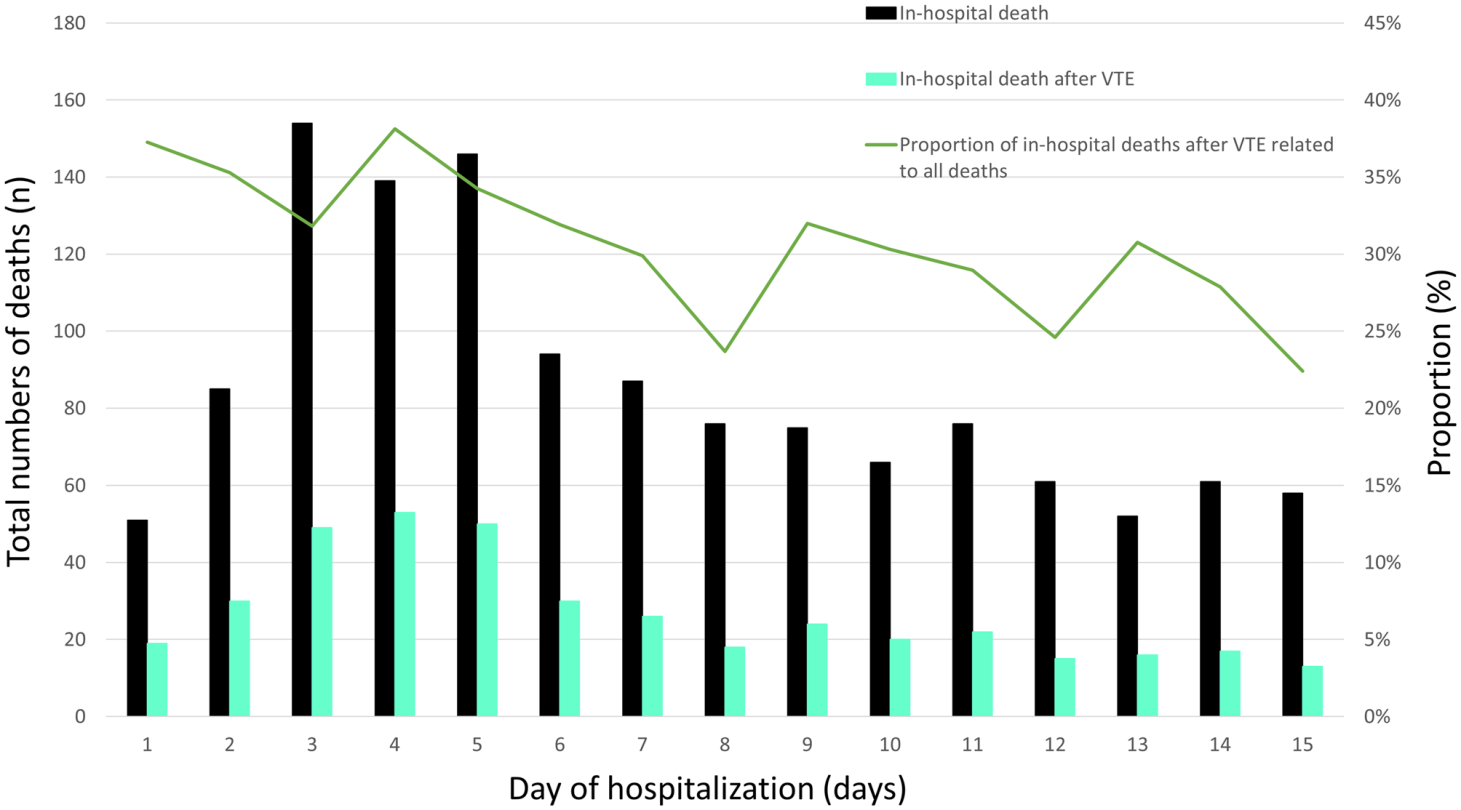
Time trend regarding cumulative number of deaths during the hospitalization period in patients undergoing elective primary KJR in Germany from 2005 to 2016. Total numbers of in-hospital deaths at the different hospitalization days (black bars), in-hospital deaths of patients undergoing elective primary KJR with VTE (green bars) and proportion of deaths in conjunction with VTE. Only the in-hospital deaths of the first 15 days after admission were taken into account.

In patients undergoing primary KJR, the risk of death was 22-fold higher if they suffered VTE compared to patients without VTE (2.2% vs. 0.1%, P < 0.001) (Table 1). In the logistic regression models, VTE increased the risk of dying during hospitalization univariably (OR 25.65 [95% CI 23.22–28.34], P < 0.001), but also independently from age, sex and comorbidities (OR 20.86 [95% CI 18.78–23.15], P < 0.001). The impact of VTE on in-hospital varied annually (Table S3 in the supplementary material). In addition, we analysed the total numbers of in-hospital deaths in patients undergoing primary KJR at each day of hospitalization (day 1–15) and the proportion of deaths, which were related to prior VTE events during hospitalization at each day. The proportion of VTE related death was highest at the first 7 days after admission and decreased over the hospitalization period (Fig. 3). As expected, bleeding complications such as intracerebral and gastrointestinal bleeding as well as transfusions of blood components were more often detected in KJR patients with VTE (Table 1). The rate of VTE and bleeding complications remained low and decreased over time, as did the need for transfusions of blood constituents (Figs. 2C and 3C as well as Table S2 in the supplementary material). The length of in-hospital stay was in median 12.0 days (IQR 10.0–14.0) and was longer in patients with (14 [12–18]) vs. without (12 [10–14] days) VTE (P < 0.001; Table 1). VTE occurrence was associated with hospitalization longer than 14 days (crude: OR 3.09 [95% CI 3.01–3.18], P < 0.001]; adjusted model: OR 3.09 [95% CI 3.00–3.18], P < 0.001). This result remained stable after additional adjustment for the year of hospitalization (OR 3.11 [95% CI 3.02–3.20], P < 0.001). Nevertheless, the impact of VTE regarding prolonged hospitalization increased slightly over time (Table S4 in the supplementary material).

## Discussion

VTE, with its clinical manifestations of DVT and PE, is responsible for significant morbidity and mortality in Europe and worldwide^5,12^. Important major provoking risk factors of VTE are represented by orthopaedic-related issues, notably major trauma and surgery, lower-limb fracture, joint replacement, and spinal cord injury^5^.

Our study results demonstrate an increasing demand and rising annual numbers of performed KJR in the aging western populations, in line with what described in other high-income states^2,6,8–11^. These temporal trends regarding total numbers of KJR and perioperative complications are important for adequate management of public health as well as health care service planning. Although KJR can substantially improve patients` mobility and quality of life in patients with advanced gonarthritis, it is accompanied by a high risk of VTE^2,13–16^. While without thromboprophylaxis, the rate of DVT in screening examinations in patients undergoing KJR was reported as approximately 60% (including symptomatic as well as asymptomatic DVT events)^13,14^, the risk of VTE after KJR can be significantly decreased by the use of pharmacological thromboprophylaxis^2^. In accordance with results from studies of KJR conducted in other states reporting a 1.1% in-hospital VTE rate after KJR^17^, we identified an in-hospital VTE rate of 1.3% in patients undergoing elective KJR in Germany. Importantly for adequate health care planning, the VTE rate decreased significantly from 1.9% in 2005 to 0.9% in 2016. This favorable trend may be due to several factors, including the introduction of the direct oral anticoagulants, the decrease in the use of tourniquets and/or surgical time of tourniquet use, a small increase in the number of uncemented KJR procedures^18^, the incline in unicondylar KJR^18^, decreasing age and the introduction of fast-track procedures improving early mobilization and discharge^19,20^.

Current practice guidelines provide partly conflicting recommendations for the medical VTE prophylaxis after KJR^21,22^. Additionally, the appropriate duration of anticoagulation following elective KJR is highly controversial and a dynamically evolving topic. Country-specific guideline recommendations as well as physicians’ and patients’ preferences, have a major impact on the management strategies. While the German AWMF guidelines recommend low-molecular weight heparins (LMWH), fondaparinux or novel oral anticoagulants (NOAC) at a prophylactic dose for 11–14 days after KJR^22^, the National Institute of Health and Care Excellence guidelines (NICE) in the United Kingdom recommend LMWH, or NOACs, with aspirin recommended for extended prophylaxis^21,23^. The guidelines of the American College of Chest Physicians and the American Association of Orthopaedic Surgeons recommend LMWH, (N)OACs or aspirin^3,6,7,21,24^.

There is still a great controversy about the effect of tourniquets on development of VTE after KJR^25–28^. The use of a tourniquet improves visualization during KJR surgery and may shorten the operating time^28^. However, there is also some evidence that the use of tourniquet during KJR surgery may lead to increased numbers of DVT^25–28^. Furthermore, previous studies have reported that cemented fixation as compared to cement-less fixation was found as a risk factor for VTE in hip and KJR^29,30^; nevertheless, the role of cement as a thrombogenic agent and cause of emboli during or after joint replacements is not entirely clear to date^30–32^. In Germany more than 90% of the KJR were operated with cement fixation^18^. Finally, the decreasing proportion of VTE in KJR patients may partly be explained by trends regarding patients’ age during the observational period. It is known that the incidence of VTE grows exponentially with age^33–35^. The proportion of patients aged ≥ 70 years decreased from 2005 to 2016, and this may have contributed to the decrease of the proportion of VTE events in KJR.

We confirmed the role of relevant predictors of VTE, such as age^19,36,37^, cancer^37^, female gender^38^, obesity^37,39^ as well as heart failure^37,40^, AF^41^, peripheral artery disease^42^, COPD^43,44^, and renal insufficiency^45^. In addition, acute cardiovascular events during hospitalization for elective KJR such as myocardial infarction^40,46^ and stroke^37,47^, but also infections^48^ occurring during hospitalization were significantly associated with VTE development, although our study design does not permit speculation about cause and effect. In fact, our results demonstrated that systemic infectious diseases (pneumonia and sepsis) were accompanied with a higher VTE risk than traditional VTE risk factors like cancer and thrombophilia. VTE events in patients undergoing primary KJR were associated with a significant 3.1-fold risk of prolonged hospitalization (beyond 14 days).

Our study highlights that perioperative VTE events aggravate the early prognosis of patients operated at the knee joint: perioperative VTE events were associated with a 21-fold increased risk of in-hospital deaths independently from age, sex and comorbidities. In parallel with reduction of VTE events, the KJR patients’ in-hospital mortality decreased from 2005 to 2016. In addition, we detected a decreasing rate of periprocedural myocardial infarction, stroke and major bleeding, which might have contributed to this mortality reduction.

Although the median duration of hospitalization for elective KJR decreased from 15 days in the year 2005 to 10 days in the year 2016, the median length of stay in the year 2016 was still substantially longer in Germany than in the United Kingdom (6 days in regular medical care and 3 days for enhanced recovery programs)^49^ or in the United States (4 days)^50^. In other European countries, enhanced recovery protocols have been adopted, resulting in short length of hospitalization of only 2 days (in median)^20^. Interestingly, enhanced recovery with halved length of in-hospital stay was accompanied by lower rate of cardiac ischemic events and lower mortality rate at 30 and 90 days follow-up after KJR and hip joint replacement^49^.

Recent studies of nationwide cohorts reported that patients treated according these protocols had a low 90-day VTE rate of 0.39%^20^. The results of these mentioned studies contributed to fuel the discussion on whether prolonged pharmacological thromboprophylaxis (after discharge from hospital) is beneficial and needed for all patients treated with an enhanced recovery protocol and hospitalized for a period of 5 days or shorter^19,20^. In these patients, a preventive strategy based on in-hospital thrombo-prophylactic treatment with the Factor Xa inhibitor rivaroxaban followed by low-dose aspirin appeared to be effective and safe for thromboprophylaxis^50^. These results remain to be confirmed in other countries, in which fast-track surgery and enhanced recovery protocols will be introduced or were introduced more recently^21^.

The key strength of our present analysis of the German nationwide inpatient sample is the very large number of unselected patients undergoing KJR, presenting real-world and practice-based data without selection bias. On the other hand, since our study results are based on ICD discharge codes and OPS diagnostic, procedural and/or surgical coding, it has to be mentioned that misclassification of clinical events, underestimation of event rates driven by underreporting or undercoding by the hospital personnel cannot be excluded. Additionally, we could not analyze any possible repeated hospitalizations of the same patient. A further limitation of our study is the focus on the in-hospital period without assessment of events after discharge, which may also potentially lead to an underestimate of the total number of postoperative VTE events. Due to coding reasons, we were not able to distinguish between unilateral and bilateral KJR. Nevertheless, it has been published that the proportion of bilateral KJR during one hospitalization is < 4% in Germany^51^. Neither prior VTE, nor anticoagulant, antiplatelet treatment or tranexamic acid use were assessed in the German nationwide inpatient sample. Thus, the potential impact of these factors on occurrence of VTE after KJR could not be analyzed, which has to be mentioned as a further limitation of our study^52^. As per routine clinical practice, venous ultrasound to diagnose DVT was primarily performed in symptomatic patients. It must be considered that the results from the German nationwide sample may not be generalizable for other geographic regions, countries, and health systems.

In conclusion, perioperative VTE was recorded in 1.3% of the KJR patients during hospitalization. While the number of KJR increased in Germany from 2005 to 2016, the rate of VTE events decreased from 1.9% in the year 2005 to 0.9% in 2016. VTE events in patients undergoing primary elective KJR were associated with a 21-fold risk of in-hospital death rates and a 3.1-fold risk for prolonged hospitalization.

## Patients and methods

### Data source

The analyses of our study were run on our behalf by the Research Data Center (RDC) of the Federal Statistical Office and the Statistical Offices of the federal states in Wiesbaden, Germany (source: RDC of the Federal Statistical Office and the Statistical Offices of the federal states, DRG Statistics 2005–2016, own calculations). Priorly, we had provided the created SPSS scripts to the RDC. After computing the analyses by the RDC, we received the aggregated results of the statistics (SPSS software, version 20.0, SPSS Inc., Chicago, Illinois). For this study, we selected and included all surgical patients with elective primary KJR hospitalized in the timeframe between January 2005 and December 2016 in German hospitals. Since we intended to focus only on hospitalized patients with elective primary procedures, we excluded patients with acute fractures. The study flow was described in part previously^53–56^. The study results are not part of the routine work of the RDC, but is an analysis especially done for our needs and proposed contents (see Fig. S1 in the supplementary material for study flow chart).

### Ethical aspects and study oversight

As described above, the investigators had no access to data of individual patients, but only to aggregated results provided by the RDC. Thus, approval by an ethics committee and patients’ informed consent were not required, in accordance with German law. The study was prepared according to STROBE recommendations.

### Diagnosis and procedural codes

In the year 2004, diagnosis- and procedure-related remuneration system was introduced in Germany with the German Diagnosis Related Groups (G-DRG) system. Data about patients’ diagnoses were gathered by the RDC of the Federal Statistical Office and the Statistical Offices of the federal states in Wiesbaden (Germany). The diagnoses are coded according to the International Classification of Diseases and Related Health Problems with German Modification (10th Revision with German Modification-ICD-10-GM), and the performed patients’ diagnostic, surgical and interventional procedures are coded according to the German Procedure Classification (OPS, surgery and procedures codes [Operationen- und Prozedurenschlüssel])^35,52^.

In our study, we included all hospitalizations, which refer to surgical patients undergoing elective primary KJR in Germany from the year 2005 to the year 2016. All hospitalized patients with primary KJR were identified based on the OPS code 5–822, after exclusion of patients with revision-surgery or replacement of existing prostheses (OPS-code 5–823) as well as acute fractures (excluding patients with distal femur fracture (ICD-code S72.4) and proximal tibia fracture (ICD-code S82.1)^52^.

### Study outcomes

The outcomes of this study were defined as VTE (including deep venous thrombosis or thrombophlebitis (DVT, ICD codes I80, I81, I82) and/or PE (ICD code I26)), prolonged hospitalization (of more than 14 days) and death from any causes, respectively recorded during hospital stay.

### Statistical methods

Descriptive statistics for patient characteristics of KJR patients with and without perioperative VTE were provided as median and interquartile range (IQR) or absolute numbers and corresponding percentages. The Mann–Whitney-U test was used to test the continuous variables of the groups regarding differences and categorical variables were compared with chi^2^ test or Fisher’s exact test, as appropriate^52^.

We analysed total numbers and incidence of VTE events in elective KJR in Germany between the years 2005 and 2016 and tested for temporal trends of VTE incidence and in-hospital death rate using linear regression models. Results are presented as estimated slope beta (β)-estimates and corresponding 95% confidence intervals (CI)^35^.

In addition, we analysed univariate and multivariable logistic regression models in order to detect influences regarding possible predictors of VTE events. Furthermore, we analysed the association between VTE and in-hospital death as well as VTE and prolonged hospitalization (of more than 14 days). These results of our study are presented as odds ratio (OR) and 95% CI. Multivariable regression models were adjusted for age, sex, obesity, cancer, coronary artery disease, heart failure, atrial fibrillation/flutter (AF), essential arterial hypertension, chronic obstructive pulmonary disease (COPD), acute and chronic renal failure, and diabetes mellitus. These covariates were selected a priori since they represent known risk factors for VTE and in-hospital death. The software SPSS (version 20.0; SPSS Inc., Chicago, Illinois) was used for computerised analysis. P values of < 0.05 (two-sided) were considered to be statistically significant^53–56^.

## Supplementary information

Supplementary Information.

## Abbreviations

AF: Atrial fibrillation/flutter
CI: Confidence interval
COPD: Chronic obstructive pulmonary disease
DRG: Diagnosis related groups
DVT: Deep venous thrombosis or thrombophlebitis
ICD: International Classification of Diseases and Related Health Problems
IQR: Interquartile range
KJR: Knee joint replacement
LMWH: Low-molecular weight heparins
NOAC: Novel oral anticoagulant
OAC: Oral anticoagulant
OPS: Diagnostic, surgery and interventional procedures codes (Operationen- und Prozedurenschlüssel)
OR: Odds ratio
PE: Pulmonary embolism
RDC: Research data center
VTE: Venous thromboembolism
