# Supplementary material for: Venous thromboembolism in patients hospitalized for knee joint replacement surgery
Source: Sci Rep. 2020 Dec 31;10:22440. doi: 10.1038/s41598-020-79490-w (PMC7775461; doi:10.1038/s41598-020-79490-w)
Supplement: Supplementary file 1 — Supplementary Information. [file 41598_2020_79490_MOESM1_ESM.docx]

**Supplementary material**

**Venous Thromboembolism in Patients Hospitalized for Knee Joint Replacement Surgery**

Short title: Keller K et al.: VTE in Knee Joint Replacement Surgery

Karsten Keller, MD^1,2*^; Lukas Hobohm, MD^1,2*^; Stefano Barco, MD^2,3^; Irene Schmidtmann, PhD^4^; Thomas Münzel, MD^1,5^; Martin Engelhardt, MD^6,7^; Lukas Eckhard, MD^8^; Stavros V. Konstantinides, MD^2,9^; Philipp Drees, MD^8^

^1^ Center for Cardiology, Cardiology I, University Medical Center Mainz (Johannes Gutenberg-University Mainz), Mainz, Germany

^2^ Center for Thrombosis and Hemostasis (CTH), University Medical Center Mainz (Johannes Gutenberg-University Mainz), Mainz, Germany

^3^ Clinic for Angiology, University Hospital Zurich, Zurich, Switzerland

^4^ Institute of Medical Biostatistics, Epidemiology and Informatics (IMBEI), University Medical Center Mainz (Johannes Gutenberg-University Mainz), Mainz, Germany

^5^ German Center for Cardiovascular Research (DZHK), Partner Site Rhine Main, Germany

^6^ Department for Orthopaedics, Trauma Surgery and Hand Surgery, Klinikum Osnabrück, Osnabrück, Germany

^7^ Institute for Applied Training Science Leipzig, Germany

^8^ Department of Orthopaedics and Traumatology, University Medical Center Mainz (Johannes Gutenberg-University Mainz), Mainz, Germany

^9^ Department of Cardiology, Democritus University of Thrace, Alexandroupolis, Greece

* K.K. und L.H. contributed equally and shared first authorship.

***Funding****.* This study was supported by the German Federal Ministry of Education and Research (BMBF 01EO1503), institutional grant for the Center for Thrombosis and Hemostasis. The authors are responsible for the contents of this publication.

**Correspondence:**

Karsten Keller, MD, Center for Cardiology, Cardiology I, University Medical Center Mainz, Johannes Gutenberg-University Mainz, Langenbeckstr. 1, 55131 Mainz, Germany, Telephone: 0049-6131-17-8380; Telefax: 0049-6131-17-8461, E-Mail: Karsten.Keller@unimedizin-mainz.de

Abstract: word count: 194 words

Paper: word count: 3083 words (main text body); 2 Figures; 2 Tables

Supplementary material: 434 words; 2 Figures; 2 Tables

**Supplementary material**

**Results**

**Figure S1:** Flowchart


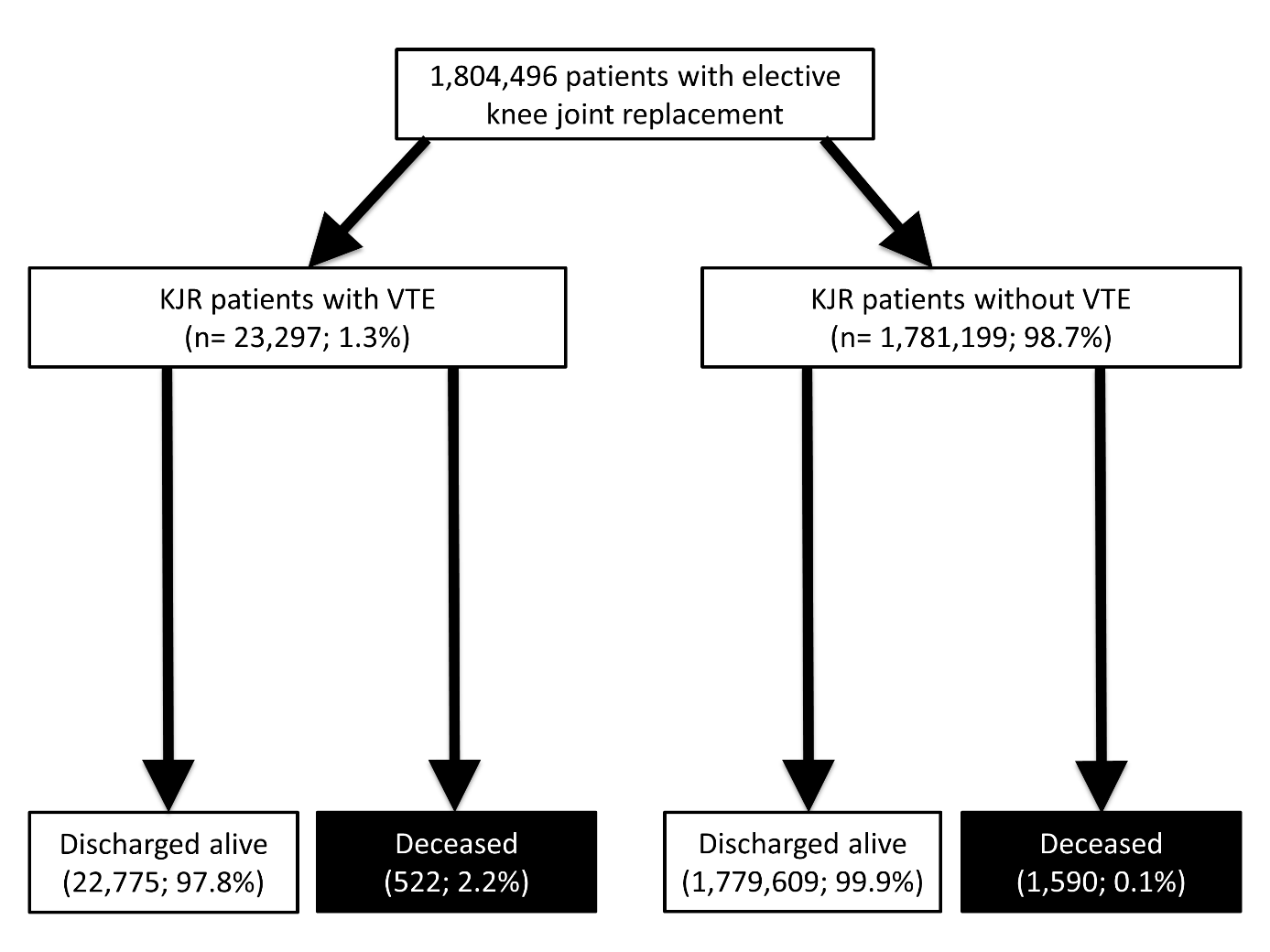


**Figure S2:** Temporal trends regarding sex, older age and comorbidities of patients undergoing elective primary knee joint replacement surgery 2005-2016 in Germany

**Panel A**: Annual percentages of female (pink bars) and male (blue bars) patients

**Panel B**: Annual rate of patients undergoing elective primary knee joint replacement surgery ≥70 years (blue bars)

**Panel C**: Percentage of female (pink bars) and male (blue bars) patients in the different age-decades


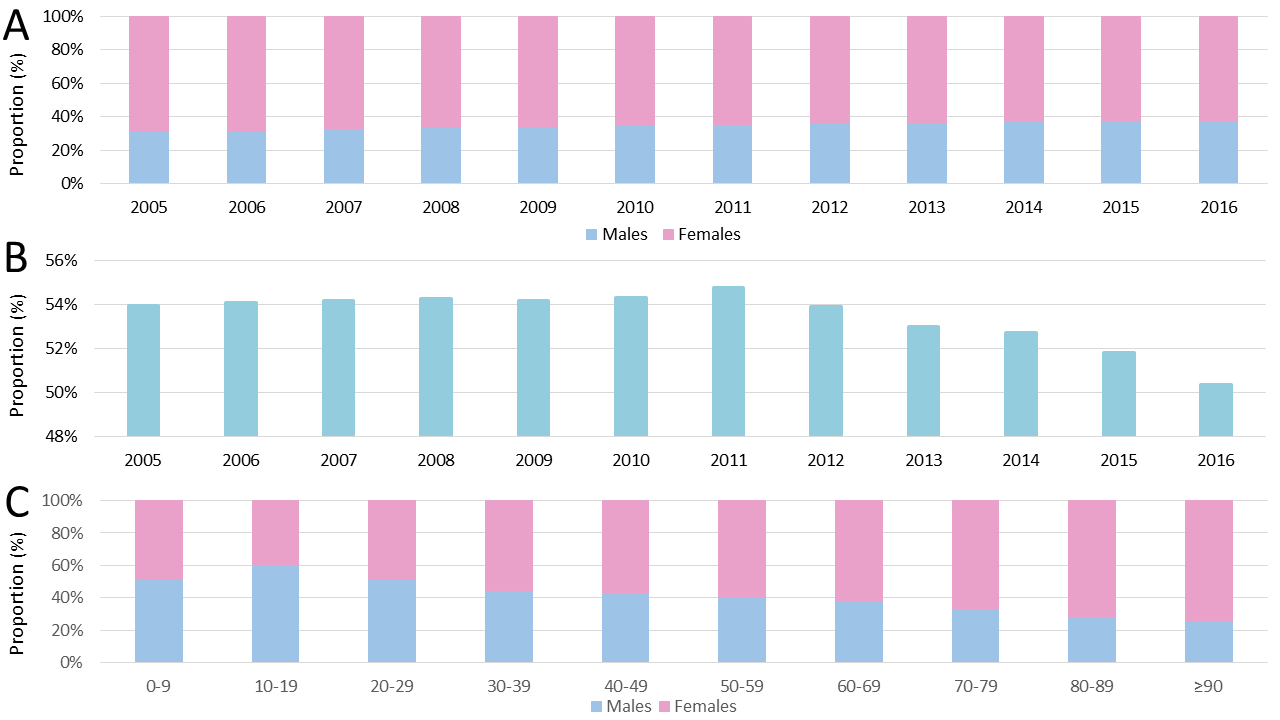


**Figure S3:** Median duration of hospitalization (solid black line) with 25% and 75% IQR (dashed black lines) for each age-decade of life over the entire period 2005-2016 (cumulative data).

**Figure S4:** Annual rate of comorbidities in patients undergoing elective primary knee joint replacement surgery

Proportion (%)

**Figure S5:** Percentage of patients developing infectious complications such as sepsis (red bars), pneumonia (green bars) and urinary tract infection (orange bars) out of all patients undergoing elective KJR.

**Table S1: Annual time trends regarding patient characteristics** **of patients undergoing elective primary knee joint replacement surgery 2005-2016 in Germany**

|  | **2005** | **2016** | **β-estimate (95% CI)** | **P-value** |
| --- | --- | --- | --- | --- |
| Age ≥70 years | 54.0% | 50.3% | -0.14 (-0.15 to -0.13) | **<0.001** |
| Female gender | 68.9% | 62.2% | -0.32 (-0.33 to -0.31) | **<0.001** |
| In-hospital stay (days) | 15 (13-16) | 10 (8-12) | -0.12 (-0.12 to -0.12) | **<0.001** |
| Obesity | 26.4% | 25.6% | 0.013 (0.001 to 0.025) | **0.028** |
| **Comorbidities** | | | | |
| Cancer | 1.2% | 0.6% | -0.70 (-0.75 to -0.64) | **<0.001** |
| Coronary artery disease | 11.2% | 8.2% | -0.39 (-0.41 to -0.37) | **<0.001** |
| Chronic heart failure | 7.9% | 4.2% | 0.15 (0.10 to 0.21) | **<0.001** |
| Peripheral artery disease | 0.85% | 0.92% | 0.45 (0.44 to 0.47) | **<0.001** |
| Atrial fibrillation/flutter | 4.3% | 7.4% | 0.62 (0.60 to 0.64) | **<0.001** |
| Chronic obstructive pulmonary disease | 3.5% | 3.5% | 0.34 (0.01 to 0.61) | **0.015** |
| Essential arterial hypertension | 52.8% | 63.7% | 0.50 (0.49 to 0.51) | **<0.001** |
| Acute and chronic renal failure | 2.9% | 8.0% | 1.05 (1.02 to 1.07) | **<0.001** |
| Diabetes mellitus | 15.1% | 17.6% | 0.19 (0.18 to 0.21) | **<0.001** |

**Table S2: Annual time trends regarding in-hospital events** **of patients undergoing elective primary knee joint replacement surgery 2005-2016 in Germany**

|  | **2005** | **2016** | **β-estimate (95% CI)** | **P-value** |
| --- | --- | --- | --- | --- |
| In-hospital death | 0.14% | 0.09% | -0.44 (-0.59 to -0.30) | **<0.001** |
| Pneumonia | 0.4% | 0.3% | 0.14 (0.05 to 0.23) | **<0.001** |
| Sepsis | 0.03% | 0.05% | 0.46 (0.21 to 0.71) | **<0.001** |
| Urinary tract infection | 3.0% | 2.5% | -0.12 (-0.15 to -0.09) | **<0.001** |
| Venous thromboembolism | 1.9% | 0.9% | -0.77 (-0.81 to -0.72) | **<0.001** |
| Deep venous thrombosis or thrombophlebitis | 1.6% | 0.8% | -0.82 (-0.87 to -0.77) | **<0.001** |
| Pulmonary embolism | 0.4% | 0.2% | -0.52 (-0.61 to -0.42) | **<0.001** |
| Unstable pulmonary embolism | 0.03% | 0.03% | -0.20 (-0.48 to 0.09) | 0.175 |
| Shock | 0.1% | 0.2% | 0.73 (0.59 to 0.87) | **<0.001** |
| Cardio-pulmonary resuscitation | 0.12% | 0.11% | -0.12 (-0.26 to 0.14) | 0.078 |
| Myocardial infarction | 0.19% | 0.15% | -0.24 (-0.36 to -0.13) | **<0.001** |
| Stroke | 0.3% | 0.1% | -0.89 (-1.01 to -0.76) | **<0.001** |
| Intracerebral bleeding | 0.011% | 0.002% | -0.88 (-1.50 to -0.26) | **0.005** |
| Gastro-intestinal bleeding | 0.11% | 0.10% | 0.07 (-0.09 to 0.23) | 0.368 |
| Transfusion of blood constituents | 13.8% | 6.6% | -0.73 (-0.74 to -0.71) | **<0.001** |

**Table S3**: Impact of VTE on in-hospital death of patients undergoing elective primary knee joint replacement surgery in Germany annually 2005-2016 (univariate and multivariate logistic regression models)

| **Year** | **Univariate regression model** | | **Multivariate regression model*** | |
| --- | --- | --- | --- | --- |
|  | **OR (95% CI)** | **P-value** | **OR (95% CI)** | **P-value** |
| 2005 | 18.87 (13.54-26.28) | **<0.001** | 15.94 (11.24-22.60) | **<0.001** |
| 2006 | 21.67 (15.71-29.91) | **<0.001** | 20.18 (14.41-28.24) | **<0.001** |
| 2007 | 23.11 (16.72-31.94) | **<0.001** | 20.37 (14.52-28.58) | **<0.001** |
| 2008 | 19.38 (13.93-26.96) | **<0.001** | 17.97 (12.77-25.30) | **<0.001** |
| 2009 | 32.08 (23.55-43.68) | **<0.001** | 30.05 (21.71-41.60) | **<0.001** |
| 2010 | 23.50 (16.75-32.97) | **<0.001** | 17.98 (12.60-25.64) | **<0.001** |
| 2011 | 41.37 (30.13-56.79) | **<0.001** | 35.73 (25.67-49.74) | **<0.001** |
| 2012 | 18.85 (12.31-28.85) | **<0.001** | 13.86 (8.88-21.61) | **<0.001** |
| 2013 | 23.62 (15.89-35.10) | **<0.001** | 18.28 (12.00-27.85) | **<0.001** |
| 2014 | 24.59 (16.21-37.32) | **<0.001** | 17.90 (11.54-27.78) | **<0.001** |
| 2015 | 32.44 (23.10-45.57) | **<0.001** | 19.56 (13.54-28.25) | **<0.001** |
| 2016 | 31.56 (21.48-46.38) | **<0.001** | 20.20 (13.33-30.60) | **<0.001** |

^*^ Adjusted for age, sex, obesity, cancer, coronary artery disease, heart failure, atrial fibrillation/flutter, arterial hypertension, chronic obstructive pulmonary disease, acute and chronic renal failure, and diabetes mellitus.

**Table S4**: Association of VTE on prolonged hospitalization (longer than 14 days) of patients undergoing elective primary knee joint replacement surgery in Germany annually 2005-2016 (univariate and multivariate logistic regression models)

| **Year** | **Univariate regression model** | | **Multivariate regression model*** | |
| --- | --- | --- | --- | --- |
|  | **OR (95% CI)** | **P-value** | **OR (95% CI)** | **P-value** |
| 2005 | 2.38 (2.18-2.60) | **<0.001** | 2.24 (2.05-2.45) | **<0.001** |
| 2006 | 2.50 (2.30-2.72) | **<0.001** | 2.37 (2.18-2.59) | **<0.001** |
| 2007 | 2.55 (2.34-2.77) | **<0.001** | 2.39 (2.20-2.60) | **<0.001** |
| 2008 | 2.79 (2.56-3.03) | **<0.001** | 2.64 (2.42-2.87) | **<0.001** |
| 2009 | 3.17 (2.91-3.45) | **<0.001** | 2.99 (2.74-3.26) | **<0.001** |
| 2010 | 3.31 (3.03-3.62) | **<0.001** | 3.05 (2.78-3.34) | **<0.001** |
| 2011 | 3.74 (3.41-4.10) | **<0.001** | 3.55 (3.23-3.90) | **<0.001** |
| 2012 | 3.90 (3.54-4.29) | **<0.001** | 3.62 (3.27-4.00) | **<0.001** |
| 2013 | 4.39 (3.95-4.87) | **<0.001** | 4.10 (3.68-4.57) | **<0.001** |
| 2014 | 4.46 (4.01-4.95) | **<0.001** | 4.10 (3.68-4.58) | **<0.001** |
| 2015 | 5.09 (4.60-5.64) | **<0.001** | 4.48 (4.02-4.99) | **<0.001** |
| 2016 | 4.99 (4.48-5.55) | **<0.001** | 4.51 (4.03-5.05) | **<0.001** |

^*^ Adjusted for age, sex, obesity, cancer, coronary artery disease, heart failure, atrial fibrillation/flutter, arterial hypertension, chronic obstructive pulmonary disease, acute and chronic renal failure, and diabetes mellitus.
